# Dislocation of the Humerus

**Published:** 1874-01-01

**Authors:** Edwin Powell


### DISLOCATION OF THE HUMERUS.

CLINICAL LECTURE, SURGICAL WARDS COOK COUFTY HOSPITAL. SERVICE PROF. EDWIN POWELL, DEC. 5, 1873.

*Reported by D. A. K. Steele, M.D., Home Surgeon.*

GENTLEMEN: This patient, J. R, aged thirty-two, native of Ireland, came into the hospital Dec. 3d; states that two days previous to his admission he was pitched out of a saloon, striking on his hands and right shoulder; has had some pain in the hand and arm since; says fingers feel numb; cannot move the injured arm freely. Now let us proceed to examine him, and see if our diagnosis corresponds with that on the House-Surgeon's record. In the first place, note the general appearance of the man. Rather under medium height, somewhat round-shouldered; but in particular, you will notice a lack of symmetry in the form of the shoulders: the right acromion process is much more prominent than the left, and you will see a little depression beneath it. The natural rounded contour of the shoulder is obliterated. You also notice that the right elbow is carried away from the side of the body: does not hang down perpendicularly. The line of axis of the humerus is directed towards the axilla, instead of towards the glenoid cavity. By palpation we detect a rounded body in the axilla. Now, as to posture, let us apply Professor Dugas' test. He states that, "If the fingers of the injured limb can be placed, by the patient or by the surgeon, upon the sound shoulder, while the elbow touches the thorax, there can be no dislocation; and if this cannot be done, there must be a dislocation." In other words, it is physically impossible to bring the elbow in contact with the sternum, or front of the thorax, if there be a dislocation; and the inability to do this, is proof positive of the existence of a dislocation, inasmuch as no other injury of the shoulder-joint can induce this disability. This is a little more positive language than I think a surgeon is justified in using on any surgical subject. But Professor Dugas is one of those men who love to use positive statements. Now, let us see: As I put the injured hand on the sound shoulder, you see the elbow recedes from the sternum. This is caused by the anatomical structure of the parts. The chest being an elliptical body, and the humerus straight, if its head be locked in behind the acromial end of the clavicle it is impossible to bring the elbow down on the thorax without using undue force. We have also, as you see, an absence of crepitus on rotation.

In a case as clearly marked as this, it would seem that it would be impossible to make a mistake in the diagnosis; and yet occasionally mistakes are made, as you have seen in one of our recent clinics. When the parts are contused and considerably swollen around the joint, many of these diagnostic marks may be rendered obscure; but in a case where the symptoms are as clearly marked as this, you would never be justified in making a mistake. There are three different forms of dislocation of the shoulder-joint: downwards, or subglenoid, as in this case; forward, or subcorocoid; and backward, or subspinous, a rare form of dislocation. I am glad to see that Professor Hamilton, in his recent work on Surgery, has discovered the idea of a partial traumatic luxation of the head of the humerus, as described by Sir Astley Cooper. I could never bring myself to understand how, under any circumstances, such a luxation could occur, from the anatomical conformation of the parts. The only way, in my opinion, in which we could get a partial luxation, would be as the result of a chronic rheumatic arthritis, or muscular rheumatism. As to the method of reduction, there are a variety of ways employed: First, by manipulation, with the arm at right angles with the body; second, with the knee in the axilla as a fulcrum, using the humerus as a lever; third, extension with the heel in the axilla; fourth, extension and counter-extension, as used by Nathan R. Smith.

At this point the patient was etherized, and Professor Powell effected the reduction by means of extension and pressure in the axilla, the head of the humerus being wedged below the acromial end of the clavicle. The arm was then dressed in a sling, and the patient directed to use it gradually.

During the remainder of the clinic hour, the attention of the class was directed to a case of concussion of the brain, from a fall; a case of surgical shock; and an amputation of the thigh, lower-third, for a compound dislocation of the knee and ankle.

